# The Teeth and Orad Cavity of Pregnant Women

**Published:** 1890-04

**Authors:** 


ARTICLE V.

### THE TEETH AND ORAD CAVITY OF PREGNANT WOMEN.

In an article on the teeth and oral cavity of pregnant women, in the *Journal of the American Medical Association*, Feb. 21, 1890, Dr. John S. Marshall says: From time almost immemorial, pregnancy has been recognized as having a deleterious effect upon the teeth; and this opinion has become so general that it has been crystallized into the familiar adage—"For every child a tooth." All writers upon obstetrics and the care of pregnant women recognize the fact that during the periods of gestation and lactation women are more liable to suffer from diseases of the teeth and neuralgia of the facial region than at other times; but the causes, prevention or mitigation of these diseases has received but little attention; in fact, it seems to be considered by most writers that these troubles are among the inevitable consequences of gestation and that little can be done to relieve them.

With this view I do not fully agree, for I believe much can be done to prevent or relieve the suffering incident to caries and the loss of the teeth, and the neuralgic condition of the face and mouth so common at this period. But in order to appreciate the causes and the treatment of these disorders, we must have in mind the changed conditions of the organism. The pregnant state is characterized by many and varied changes in the general system and in special organs, the most marked of which are those of the uterus, the genitals and mammary glands, but which it is not necessary for us to take time to describe, as they are generally well understood. The changes which bear the closest relationship to our subject are those of nutrition affecting the condition of the blood, the bones, the teeth and the excretions and secretions.

Pregnancy usually exercises a favorable influence upon nutrition; after the third or fourth month the appetite improves, adipose tissue is laid on and the body weight is progressively increased, so that at the completion of gestation it is about one-thirteenth greater than before. Many times, however, the reverse of this is met with; nutrition is so disturbed as to cause considerable emaciation and general debility.

An increase in the salivary secretions is often a noticeable symptom. Ptyalism, when present, manifests itself early and usually disappears spontaneously between the third and fourth months. It occasionally persists, however, in an aggravated form during the entire period of gestation, and even for several weeks after, and the amount secreted may be so great as to endanger the life of the patient. The qualitative changes in the saliva during pregnancy are sometimes quite marked. The water is increased, while the organic and inorganic elements are diminished. Schramm reported one case in which the ptyalin was entirely absent, In those cases in which excessive ptyalism is manifested the buccal mucous membrane is more or less inflamed, the parotid, submaxillary and sublingual glands are swollen and tender, and quite painful when their functions are especially excited. Fetor is not present, and the absence of this symptom distinguishes it from mercurial ptyalism. The cause of this disorder is in all probability a reflex neurosis, and it frequently reappears in successive pregnancies. Gingivitis is another common accompaniment of pregnancy, and is often present when there is no indication of salivation. Pinard is of the opinion that it is more common among multiparæ than primiparæ. This opinion is based upon seventy-five cases, of whom orty-three were of multiparæ and thirty-two of primiparæ. In the former the disorder was present thirty-one times, in the latter fourteen times. Gingivitis is characterized by redness and tumefaction of the gums, and a tendency to bleed on slight pressure or friction, as in mastication or the use of the tooth-brush; while the secretions of the mucous gland are often decidedly acid in reaction, as may be easily proved by litmus paper. Treatment should consist of saline cathartics, and astringent and antacid mouth-washes, and thorough cleanliness of the mouth and teeth.

Phagedenic pericementitis is occasionally present during pregnancy. This disorder is characterized by tumefaction of the gums, which have a purplish tint along the margins, the festoons are swollen and detached from the teeth, and bleed on the least provocation; many of the teeth are loose in their alveoli, from which pus constantly exudes, and in a majority of the cases examination of the roots of these teeth will show them to be more or less covered with calculus, the character of which is still in dispute, some claiming it is salivary, others serumal. After a little time the gums begin to recede from the necks of the teeth, and it is not by any means rare for this disease to progress so rapidly that one or more teeth are lost by the disorder before the completion of gestation. The disease usually persists after delivery, but not with such rapid progress as before.

Odontalgia is generally the result of dental caries which penetrates to the pulp and sets up an acute or chronic pulpitis, or may be caused (as recently stated by Dr. W. W. Allport) by hyperemia of the pulp, due to the augmented volume of blood, increased arterial pressure and capillary hyperemia of the upper half of the body. In proof of this statement by Dr. Allport, I would call attention to the fact that a brisk cathartic will often relieve or entirely control an attack of odontalgia, or a congestive headache.

Many women are in the habit of discarding from their aliment during pregnancy all those foods which contain an abundance of calcium salts, and restrict themselves, as nearly as possible, to a fruit diet, believing that by such practice the bones of the child will be imperfectly calcified, and thus parturition will be robbed of much of its suffering. There is no scientific evidence that such a result is obtained; while on the other hand, as in those cases affected with hyperemesis, though the child when born may be small and much emaciated, it has the appearance of being properly formed and its bones as dense as in the majority of normal pregnancies, and the fact seems to be pretty well established that the osseous framework of the fetus is formed in such cases at the expense of the maternal organism.

The principal exciting cause of caries during pregnancy, as in all other cases, is the ever-present lactic acid fermentation, but its action is greatly augmented by the changed conditions of the salivary and buccal secretions. The acids found in the secretions of the mouth as a result of the disorders incident to the pregnant state, are acetic, hydrochloric, uric and oxalic. Consequently in these cases where the dentine has been rendered abnormally soft—deficient in calcium salts—there is likely to be a very rapid breaking down of tooth structure during pregnancy and lactation.

Long tedious operations, like the restoration of form in decayed teeth with gold, are inadmissible during gestation. All operations upon the teeth at such times should be as free from pain and fatigue as is possible, from the fact that in certain cases miscarriage might be the result. Caries of the teeth can be arrested by the use of temporary fillings like Hill's Stopping, or oxy-phosphate cement, until such time as the patient is in condition to bear the nervous strain incident to large gold operations. Among the most valuable of preventive measures are a thorough and frequent use of the tooth-brush and floss silk at least three times per diem, supplemented by tooth powders and antacid mouth washes.—*Medical and Surgical Reporter.*

